# ‘Pinore’: The New Red Wine Variety Cross-Bred between ‘Pinot Noir’ and ‘Regent’ Vines

**DOI:** 10.3390/plants10122666

**Published:** 2021-12-03

**Authors:** Stanko Vršič, Klemen Vršič

**Affiliations:** 1 University Centre of Viticulture and Enology Meranovo, Faculty of Agriculture and Life Sciences, University of Maribor, Pivola 10, 2311 Hoče, Slovenia; 2 Fabijan Lab, Laboratory Services, Vosek 6e, 2231 Pernica, Slovenia; klemen.vrsic@fabijan.si

**Keywords:** grapevine, breeding, red variety, ‘Pinore’, Slovenia

## Abstract

Renewed interest in varieties that are more tolerant to diseases has emerged, which is mainly due to increased awareness by producers and consumers regarding the impact of phytochemicals in the environment. This paper describes the first Slovenian grapevine variety ‘Pinore’ crossed between the *Vitis vinifera* L. ‘Pinot Noir’ clone Mf and ‘Regent’ vines. The aim was to create an early ripening grape cultivar that has a good tolerance to biotic stress (e.g., downy and powdery mildew, botrytis) combined with the benefits of established cultivars and their intense wine colors. Some ampelographic characteristics of young shoots, mature leaves, bunches, and berries are presented, and its major agronomic traits, ripening time, grape yield, quality performances, and disease resistance were evaluated over a three-year period (2014–2017). Wine sensory analyses were performed and compared with the international variety ‘Pinot Noir’. The examined genotype showed good agronomic performance and a high wine quality as far as the content of polyphenols is concerned, especially in terms of anthocyanins and tolerance to diseases (*Ren3/9* and *Rpv3.1*); it is significantly different compared to the reference variety ‘Pinot Noir’. In terms of ampelographic characteristics, the main differences are in the number of leaf lobes, the depth of the lateral sinuses, and the content of anthocyanins in its flesh. The investigated genotype has been proposed to the Committee of new varieties in Slovenia for the variety recognition procedure, and completion of the procedure planned for the end of 2023.

## 1. Introduction

The grapevine (*Vitis vinifera* L.) is the most economically important fruit species in the World (OIV 2019); therefore, cultivar selection is a major viticultural factor for increasing the yield and quality of grapes. Successful viticulture must meet the demands of consumers and growers for good wine quality, disease and insect tolerance, and low environmental impact [1]. European agricultural policies have implemented guidelines that are focused on improving management strategies, integrating agronomic practices in vineyards (2009/128/CE Directive), and a reducing the use of pesticides through the use of more disease tolerant varieties (PiWi) in place of conventional ones. These varieties could be the most promising tool for low input and cost, a lower ecological fingerprint and time-saving viticulture due to spray reduction [2]. They are also the most effective tool for increasing organic agriculture to 25% by 2030, which is in accordance with the agreement of the European Union (EU) countries (A European Green Deal 2021).

These varieties are the result of efforts to combine the quality of traditional European varieties (*Vitis vinifera* L.) and the different resistance traits typical of American (*Vitis riparia* Mchx., *Vitis aestivalis* Mchx., *Vitis berlandieri* Planch., and *Vitis rupestris* Scheele) and Asian (*Vitis amurensis* Rupr.) species. Interspecific breeding was especially important after the massive destruction of European vineyards, which was a consequence of the invasion of serious fungal diseases and pests from the U.S. during the second half of the 19th Century, such as powdery mildew (*Erysiphe necator* Schwein.), downy mildew (*Plasmopara viticola* (Berk. & M. A. Curtis) Berl. & De Toni), or phylloxera (*Daktulosphaira vitifoliae* Fitch). Downy mildew is one of the most prevalent grapevine diseases worldwide and can lead to significant reductions in berry yield and quality [3]. Different loci (*Run1* + *Rpv1*, *Rpv3.1*, *Rpv3.2*, *Rpv10*, *Rpv12*, *Ren1*, *Ren3*, *Ren9*) that confer resistance to powdery mildew and downy mildew have been identified [4]. Resistance to grapevine downy mildew mediated with *Rpv3–1* is associated with specific host transcriptional responses and the accumulation of stilbenes [5]. Unfortunately, the offspring of these varieties often lose the stable yield and good quality traits of their European parents due to their complex polygenic base, which governs the resistance and quality of the grapes [6] and demonstrates that interspecific breeding methods are quite unsuccessful. Finally, the application of pesticides and the use of the first rootstocks that are tolerant to phylloxera, the low quality of the wines that were obtained, and the possible presence of toxic metabolites have led to hybrids having low popularity [7]. Since then, crosses have only been performed in Germany [8], Austria [9], and Hungary [10], and France has mostly halted their breeding programs after hybrids were banned [11]. In the new varieties that were obtained by crossbreeding, better disease tolerance continues to be sought after. For successful breeding purposes, however, the conservation of genetic vine resources is a priority, as this allows the appropriate selection of parent materials in the breeding process.

In 1822, an internationally significant grapevine research station was established on the estate in Vrhov dol near Maribor (today the University Centre of Viticulture and Enology Meranovo), Slovenia, the primary aim of which was to introduce new cultivars (mainly from the Rhineland) into practice. The initiator of these activities at that time was Archduke John, the great-grandson of Maria Theresa. Selection in viticulture in Slovenia began immediately after the appearance of phylloxera in 1890 and even more intensively after 1906, with the selection of Teleki rootstocks from Hungary, namely the hybrids *V. riparia × V. berlandieri* Teleki No. 4, 5, 6, 7, 8, and 9 [12]. The material of Teleki No. 4 was sent to Oppenheim in 1912, and from this material, one of the most famous rootstocks ‘SO4’ was derived. In the framework of the national grapevine breeding program, planed clonal selection began in 1954, with the selection of ‘Welschriesling’ [13]. The well-known breeder Stanko Matekovič selected the first clones of the ‘Welschriesling’ Mt 178 (today SI-11) and ‘Sauvignon Blanc’ Mt 43 (today SI-3). At that time, he also crossed the varieties ‘Madeleine Angevine’ and ‘Furmint,’ and produced the hybrid 14/2, the code of which has not changed [14]. In the 1980s, we started to test the characteristics of PiWi varieties from different breeding centres in Europe (Geisenheim, Freuburg, Geilweilerhof, Kesthely, etc.) more intensively. Five of these varieties were added to the list of varieties that could be grown in the wine-growing regions Podravje and Posavje in 2021 (*Official Gazette of Republic of Slovenia*, No. 26/21). At the same time, we also started with the classical crossbreeding of wine- and table-grape varieties. The first outcome of these was ‘Pinore’, which resulted from a cross between the ‘Pinot Noir’ clone Mf × ‘Regent’ in 1999. The mother plant ‘Pinot Noir’ has favorable characteristics and a relatively short vegetative cycle and fruity aromas, and it is one of the world’s high quality red wine varieties. This clone of ‘Pinot Noir’ was chosen because according to the OIV descriptor 204, its cluster architecture (density) is loose(OIV 2009). The second parent ‘Regent’ also has a short vegetative cycle (same as the mother plant), intense color in its berries and in the wines, and good tolerance to diseases. The main purpose of this cross was to grow a variety that would be suitable for cultivation in continental climate conditions, have a more intense color than ‘Pinot Noir’, and be less susceptible to diseases (downy mildew, powdery mildew, and botrytis). We hypothesized that some offspring from the crossbreed of these two parental lines could inherit (i) a satisfactory level of disease resistance and (ii) appropriate viticultural and oenological properties for the production of quality wine in our climate conditions.

## 2. Results and Discussion

### 2.1. Genetic and Ampelographic Characteristics

**Figure 1:**
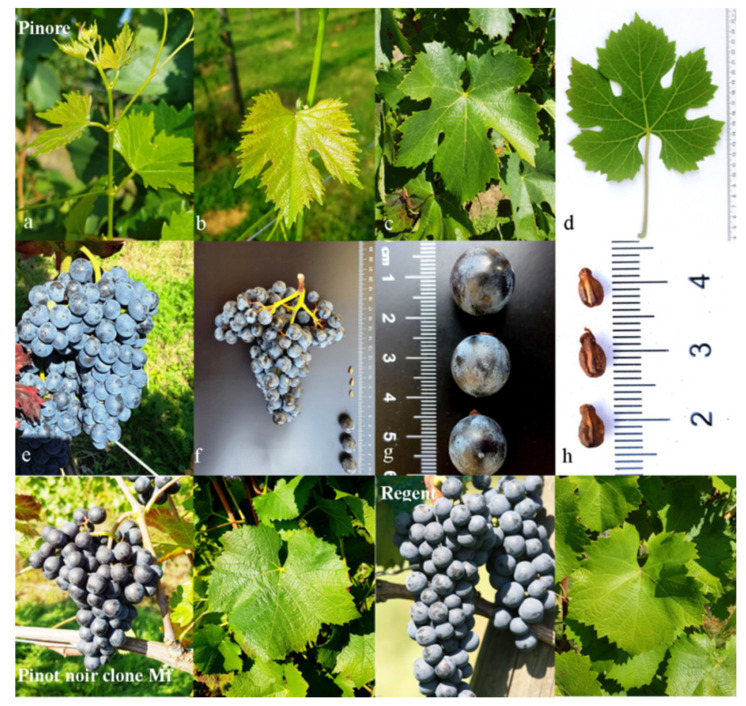
The tip (**a**) and color of upper side of leaf blade (**b**) of young shoot, mature leaf in nature (**c**) and lower side of the blade in laboratory (**d**); bunch in nature (**e**) and bunch (**f**), berries (**g**), and seeds (**h**) of the ‘Pinore’ variety in laboratory and bunch and leaf of parental varieties in nature.

The genetic profiles of the new varieties are indicated based on the analyses of nine microsatellite loci (Table 1), confirming both parental varieties (‘Regent’ and ‘Pinot Noir’). The ampelographic characteristics of the new Slovenian variety ‘Pinore’ (Figure 1) are presented in Table 2, which indicates its OIV descriptors. The tip of the young shoot is fully open, and the upper side of the leaf blade of a young leaf is yellow/bronze. The mature leaf is circular and has five lobes, and the petiole sinus is overlapped. The prostrate hairs between the main veins have a medium density, and the erect hairs on the main veins on the lower side of blade have a low density. The bunch is cylindrical, short, and is of a medium density. The berries are small, globose, and blue black, and the flesh has weak anthocyanin coloration. The main differences according to the OIV descriptors between the new variety ‘Pinore’ and ‘Pinot Noir’ are in the number of lobes (OIV68), the degree of opening of the petiole sinus (OIV079) (Table 2), and the anthocyanins in the flesh (OIV231).

### 2.2. Phenological, Agronomic, and Qualitative Performances

**Figure 2:**
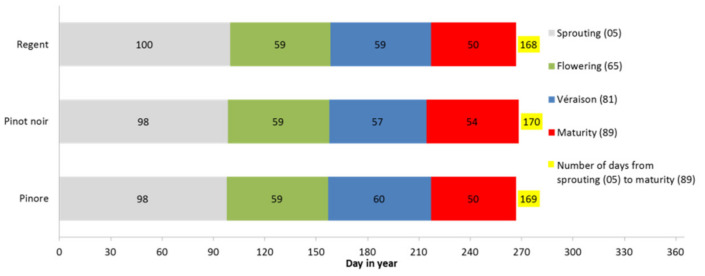
Number of days needed for the start of some phenological stages (sprouting, flowering, véraison, maturity; BBCH-scale) and number of days from budburst to maturity of ‘Pinore’ and both parental varieties.

The cultivation properties for the three-year research period showed that in the tested variety, the phenological stages (budburst, flowering, véraison, and ripening) occur during the same period as in the parental varieties (Figure 2), where the sugar content in grape juice is over the 84 °Oe, which is the minimum sugar content for top-quality wine with controlled and guaranteed geographical origin and quality (*Official Gazette of the RS*, No. 105/06). Under these conditions, it can be harvested at the beginning of the last week of September (22 September ± 3 days) in most years, depending on the environmental conditions (Table 3).

The tested variety displays good fertility, with 93 berries per cluster (Table 3) on average. Its fertility is appropriate and is not susceptible to uneven ripening. The cluster is short (OIV202), and the length of the peduncle is short (OIV206). The 3-year average weight of the cluster was 169 g (reference variety 161 g), and on our own rooted plants, it was 117 g (*p* ≤ 0.05). The berries are small (OIV 503), and the average weight of the berries on the grafted vines was 1.7 g and of 1.2 g on our own rooted plants. Berry size is considered to be of great importance to winemakers due to the belief that smaller berries make better wines [15]. Its blue-black berry is soft and juicy, with low understated color in the flesh. The berry skin is medium thick, and cracking was not observed. Its taste is neutral, with a slight herbal flavor sometimes being able to be noted. The average titratable acid (9.93 g/L) and sugar content (88.7 °Oe) of the fruit (2014–2016) was similar to that of the reference variety (Table 3).

The productivity of the tested variety is shown in Table 3. Differences in the yield per vine between ‘Pinore’ (2.51 kg vine^−1^) and ‘Pinot Noir’ (2.38 kg vine^−1^) were not significant (Table 3). Our own rooted nuclear stock of ‘Pinore’ had a one third lower yield per vine and a lower mean weight of berry than that grafted on a ‘Kober 5BB’ rootstock (*p* = 0.05), which could also be due to the pathological effects of phylloxera in our own rooted plants. This can only be inferred from the lower vigor of the plants because a phylloxera attack assessment has not been conducted. The new variety showed good compatibility with different rootstocks (data are not presented), which was at the same level as for the reference variety. In terms of bunch and berry weight and grape quality, the new variety did not surpass the reference cultivar. Its growth is moderately intensive, and canopy management during the growing season is the same as in the ‘Pinot Noir’ variety. A yield of 8 to 10 t/ha can be achieved with 8 to 10 buds per vine (mono Guyot), with approx. 4000 stocks per ha. It is primarily recommended for growth in sites that are traditionally suitable for red cultivar production.

The pathological characteristics showed that the new variety is less susceptible to downy and powdery mildew (four spraying per year) than the reference variety (8–10 spraying per year). This was also confirmed with genetic analyses (JKI, Geiweilerhof, Germany), which showed that that it has the same loci of resistance (*Ren3/9* and *Rpv3.1*) as the ‘Regent’. Natural disease resistance is a cost-effective and environmentally friendly way of controlling plant disease, but the resistance needs to be effective and durable. Vineyard trials have demonstrated that the *Rpv12/Rpv3*- and *Rpv3*-cultivars are a powerful tool to reduce the dependence of grape production on fungicide applications [3,16]. Furthermore, they report on the emergence of a new *P. viticola* isolate that is able to overcome both *Rpv3*- and *Rpv12*-mediated resistance [16,17]. Therefore, our new variety was assessed as only being less susceptible to downy mildew, its resistance is slightly lower than that of ‘Regent’, especially in weather conditions that are less favorable than those in 2014. However, this does not rule out that there could be another, untested loci present, as found in Georgian-derived accessions [18]. Three highly significant novel loci were identified on chromosomes 14 (*Rpv29*), 3 (*Rpv30*), and 16 (*Rpv31*) that were associated with a low level of pathogen sporulation. This is the first evidence of loci that are resistant against *P. viticola* in *V. vinifera* germplasm and is the first time that potential target genes for breeding *P. viticola*-resistant grapevine cultivars have been identified. Breeding for disease resistance is also a very time-consuming process (up to 25 years). A way to considerably decrease the length of the breeding process is the adoption of the marker-assisted selection approach, which allows the targeted selection of progeny harboring the resistance loci, as suggested by Eibach and Töpfer, 2015 [19].

During the investigation, the resistance level for the powdery mildew was comparable to the ‘Regent’ in our weather conditions. The new variety is not prone to bunch rot (botrytis); thus, the desired technological maturity can be achieved by carefully choosing the harvest time. This is mainly due to the lower cluster density and more airy trellis than the reference variety. The fungicide reduction of up to 60% is an ecological and economic benefit. 

### 2.3. Wine Characteristics

**Figure 3:**
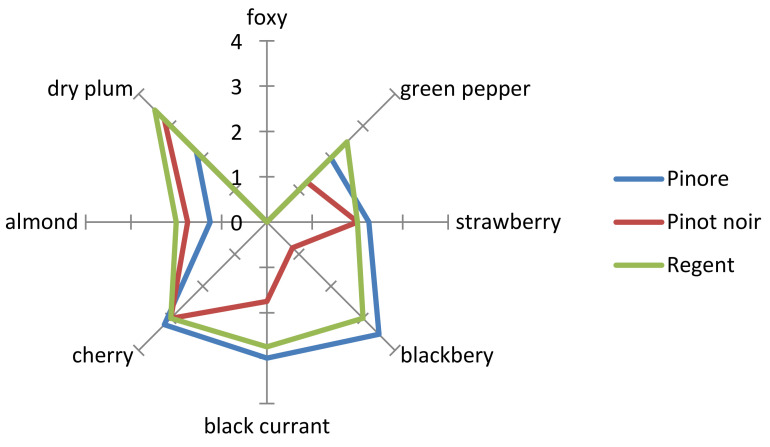
The expression of individual aromas (0–5 points) of ‘Pinore’ and ‘Pinot Noir’ varieties in sensory evaluation.

Before the start of the trial, the chemical characteristics of wine were analysed in 2013 at Mendel University, Lednice, Czech Republic (data are not presented). The results show that the tested variety had a higher content of anthocyans than both parental varieties (two times higher than ‘Regent’ and 22 times higher than ‘Pinot Noir’). The chemical composition of the grapes was variable from year to year (especially in rainy 2014). The average content of anthocyans in the wine of ‘Pinore’ (2014–2016) was 1534 mg/L (Table 4), from 570 mg/L in 2014 (year with the Huglin index 235 units below the last then-year average) to 2308 mg/L in 2015. Despite the very bad weather conditions in 2014, the content of anthocyanins in the wine of the new variety was higher than that in the wines of the standard vinifera variety ‘Cabernet Sauvignon’, as stated by the Baron and Kumšta 2012 [20] in wines of Moravia and Trentino, (233 and 341 mg/L, respectively), and by Ju et al., 2021 [21] in wine from China (356 mg/L). The ratio of the anthocyan content between the new and reference varieties was similar to that in the preliminary analysis in 2013. This also confirms the color intensity. It was higher in the wine of new variety, showing a value of 126% in comparison to the reference variety (*p* ≤ 0.05). The sugar-free extract content was high, 25.3 g/L on average, which was for 7.2% higher than it was in the case of the reference variety (Table 4). Th tartaric acid content of the examined years was higher than it was in ‘Pinot Noir’(in average for 1.18 g/L), which was 24.5% higher than it was in the reference variety (*p* ≤ 0.05). The ‘Pinore’ variety enables the production of excellently colored extract wines with a good phenolic structure and fruity aroma. The taster rated it with an average rating of 17.42, whereas ‘Pinot Noir’ achieved a score of 17.29 (Buxbaum max. 20 points), but the differences were not significant and a likeability rating (1–5 points) of 3.5 points (‘Pinot Noir’ 3.6 points). Most of the evaluators defined it by type of wine, considering it to be similar to the ‘Terrano’ variety. The expression of the individual aromas of the ‘Pinore’ and the ‘Pinot Noir’ reference varieties is shown in Figure 3. Of the primary aromas in the wine from the new variety, the most pronounced aromas were those of blackberry, dry plum, and black currant.

## 3. Materials and Methods

### 3.1. Origin of the Genotype and Study Site

The crossbreeding was carried out in 1999 in a private collection of wine and table grape varieties in Zagorci (46.5087821, 15.9773642) near Ptuj, Slovenia. This study area is a steep-slope vineyard with a 15% inclination and an exposure to the SW and was established in 2010 according to the traditional Slovenian cultivation system (inter-row: 2.4 m; per vine plant: 1 m). The training system was mono Guyot. All grapevines were grafted on *V. berlandieri × V. riparia* ‘Kober 5BB’ rootstock. The vineyard was cultivated according to the viticultural practice of integrated production with an inter-row permanent green soil cover and the use of herbicide glyphosate (1.5 L/ha) within the row (vine-strip). The study was conducted from 2014 to 2016. The average amount of precipitation (1 April to 31 October) was 841 mm in 2014, 723 mm in 2015, and 563 mm in 2016 (the long-term average from 1980–2019 is 712 mm). The average temperatures were 15.9 °C (2014), 16.6 °C (2015), and 16.3 °C (2016), and the values of the Huglin index during these years were 1736, 2057, and 2017 °C units, which are near the average of the last 10 years (16.45 °C and 1981 °C units, respectively), with an exception occurring in 2014 (Meteorological station Maribor, Slovenian Environmental Agency).

The soils are medium deep loams with a pH of about 6.5 (0.1 mol/L KCl). Based on the ammonium lactate extraction procedure, an average soil sample contains 6.5 mg soluble-P_2_O_5_-P, 26.7 mg soluble-K_2_O-K, 25 mg soluble-MgO-Mg per 100 g, and 3.61% of organic matter of air-dried soil (0–30 cm). These soil characteristics were obtained before starting the trial in April 2013. Fertilizers were not applied during the experimental period.

### 3.2. Experiment Set Up

Testing of the new variety began in 2014, at the same location as the crossing, when the vines were four years old. ‘Pinot Noir’ served as the reference cultivar. For each genotype, 30 vines were monitored and were considered as 6 replicates (5 vines per replication). In each experimental year (2014–2016), each vine was pruned to nine buds per vine. To describe the phenological plant growth stages, the BBCH system was used as follows: sprouting (05): “wool stage”, brown wool clearly visible; flowering (65): full flowering, 50% of flowerhoods fallen; véraison (81): beginning of ripening, berries begin to brighten in colour; maturity (89): berries ripe for harvest.

In 2013, before the start of the experiment, the total polyphenols were measured at Mendel University, Faculty of Horticulture, Lednice, Czech Republic, in wine from tested and parental varieties. The Folin–Ciocalteu method was used to determine the total polyphenolic compounds. All of the samples were analyzed in triplicate. The absorbance (SPECORD 210, Carl-Zeiss, Jena, Germany) was measured at λ = 750 nm against a blank. The results were expressed as the gallic acid equivalent. The determination of individual antioxidant components with HPLC-UV/VIS was performed [22].

The new crossing was also determined with SSR markers [23] and was analysed for disease-resistant loci [4]. The total genomic DNA was extracted using the NucleoSpin Plant II kit (Macherey-Nagel, Düren, Germany), and it was extracted from young leaves. The extracted DNA was quantified and was used at a working DNA concentration of 1 ng/µL. Twenty-four microsatellite loci (nine recommended by two European projects Genres081 and GrapeGen06 are in Table 1) were analyzed: *VMC1B11*, *VMC4F3.1*, *VrZAG62*, *VrZAG 67*, *VrZAG79*, *VrZAG83*, *VVIH54*, *VVIB01*, *VVIN16*, *VVIN73*, *VVIP31*, *VVIP60*, *VVIQ52*, *VVIV37*, *VVIV67*, *VVMD5*, *VVMD7*, *VVMD21*, *VVMD24*, *VVMD25*, *VVMD27*, *VVMD28*, *VVMD32,* and *VVS2*. The combinations of microsatellite loci (multiplexes) were optimized at the Julius Kühn-Institut Siebeidingen laboratory; using different labels and diverse fragment lengths allowed the multiplexing of the polymerase chain reactions (PCR) with up to four markers. The KAPA Fast Multiplex PCR Kit (2x) (Kapa Biosystems, Wilmington, MA, USA) was used to set up reaction mixtures containing master mix, 100 pmol of each primer, and ~1 ng template DNA. Amplification was performed in ABI 9700 thermal cyclers (Applied Biosystems, Foster City, CA, USA) using the following program: three min initial denaturation at 95 °C followed by 30 cycles of denaturation at 95 °C (15 s), annealing at 60 °C (30 s), and extension at 72 °C (30 s). A final extension was performed at 72 °C for seven min. Analysis of the PCR amplifications was carried out using an ABI 3130 × l genetic analyzer (Applied Biosystems, Weiterstadt). The sizes of fluorescently labeled DNA fragments were determined with the GeneMapper 5.0 software (Applied Biosystems, Foster City, CA, USA) and were based on a fluorescently labeled size marker covering the range of 75 to 500 bp. For allele sizing, the reference genotypes ‘Muscat à petit grains blanc’ and ‘Cabernet franc’ were employed. The eight resistance loci (*Run1* + *Rpv1*, *Rpv3.1*, *Rpv3.2*, *Rpv10*, *Rpv12*, *Ren1*, *Ren3*, *Ren9*) that were present were used the markers, as recommended by Röckel et al., 2021 [4]. DNA extraction and thr microsatellite and disease resistance analysis were conducted at the Julius Kühn-Institute, Institute for Grapevine Breeding Geilweilerhof, Germany.

### 3.3. Sampling and Measurements

The investigations were conducted for three years (2014–2016). The ampelographic characteristics, the compatibility with the rootstocks, the time from bud burst to technological maturity (phenology), grape yield, bunch and berry morphology, and grape and wine quality were monitored. The ampelographic characteristics of the young shoots, mature leaves, and the bunches and berries were described using the OIV descriptors (OIV 2009). The ripening time was determined on the basis of sugar content, which was determined using a refractometer to be over 84 °Oe, which is the minimum content required for top-quality wine with controlled and guaranteed geographical origin and quality (*Official Gazette of the RS*, No. 105/06). The grape yield was established by measuring the weight of all of the bunches from each vine. The bunch weight was calculated based on the average of all of the bunches that were produced by the 30 vines. The berry weight was the average value of 100 randomly selected berries. The required sugar content was was established using a refractometer, and the required total acid content was determined by titration with N/4 NaOH. Wines were produced by microvinification in 40 L stainless barrels that comprised each considered variety. Five months after bottling, the wines were analysed via the official methods of the Wine Act (*Official Gazette of the RS*, No. 105/06). Standard parameters such as alcohol, total acids, total extract, and colour intensity were measured at the University Centre of Viticulture and Enology Meranovo, Faculty of Agriculture and Life Sciences. Sensory evaluation was performed by an accredited sensory committee (nine evaluators). A Buxbaum score method (0–20 points) (*Official Gazette of the RS*, No. 32/00) was used. Wine categories were established: at least 12.1 points—table wine (wine without geographic origin); at least 14.1 points—regional wine; at least 16.1 points—quality wine with controlled geographical origin; at least 18.1 points—top-quality wine with controlled and guaranteed geographical origin and quality.

### 3.4. Statistical Analysis

Before the variance analysis, all of the parameters were tested for normality and variance homogeneity using the Levene test. For the f variance analysis, one-way ANOVAs were conducted. Mean comparisons between the new and standard varieties were conducted using Tukey’s test. The data were analysed using the software IBM SPSS Statistics (vers. 25, IBM, Incorporation, Armonk, NY, USA).

## 4. Conclusions

Grapes of the ‘Pinore’ variety, which were obtained by crossing the ‘Pinot Noir’ and Regent’ varieties, can be produced with a reduced amount of fungicides (up to 60%), which has ecological and economic benefits. The satisfactory health of the vines until harvest can be achieved with four sprayings on average. However, the disease resistance needs to be effective and durable. This is more likely with multiple resistance loci. With the use of downy mildew markers in the new variety, only the single resistance locus *Rpv3* was confirmed, for which it has already been established that individual downy mildew isolates can overcome its resistance. In our weather conditions, the resistance level for powdery mildew was comparable to that of the ‘Regent’ variety, and for downy mildew, the resistance level was slightly lower than that of the ‘Regent’ variety. The new variety is less prone to bunch rot (botrytis) because the cluster is less compact, which is similar to the ‘Pinot Noir’ Mf clone, supporting hypothesis (i). During the testing of the new variety, it proved to be suitable for a continental climate. The new variety consistently produced good-quality wines, and its quality is satisfactory, regardless of vintage. The judges noted its harmonious, full-bodied and often fruity but classic presentation. In wine, the most pronounced aromas are those of blackberry, dry plum, and black currant. Additionally, the wine produces mature tannins that are suitable for barrique aging. Therefore, the hypothesis (ii), which was determined at the beginning of the breeding period, can be confirmed. This is the first variety with an intense color that is suitable for cultivation in the continental wine-growing regions of Slovenia (Podravje, Posavje). In the case of further grapevine breeding, it will be necessary to test young seedlings for disease resistance and to then to continue to only test those that demonstrate resistance to multiple loci.

## Figures and Tables

**Table 1:** Genetic profile of new variety ’Pinore’ and both parental varieties analysed on nine microsatelite loci.

Variety	VVMD27:1	VVMD27:2	VVS2:1	VVS2:2	VVMD7:1	VVMD7:2	VVMD5:1	VVMD5:2	VrZAG62:1	VrZAG62:2	VrZAG79:1	VrZAG79:2	VVMD28:1	VVMD28:2	VVMD32:1	VVMD32:2	VVMD25:1	VVMD25:2
* ‘Pinot Noir’	186	190	137	151	239	243	230	240	188	194	239	245	218	236	240	272	239	249
‘Pinore’	190	190	137	153	239	251	240	240	194	194	245	251	218	258	240	272	239	241
* ‘Regent’	186	190	133	153	247	251	228	240	194	204	251	259	234	258	240	272	241	241

* Source: Maul, E.; Röckel, F. 2015: "Regent and Pinot Noir" Vitis International Variety Catalogue (www.vivc.de, accessed on 15 October 2021).

**Table 2:** Ampelographic characteristics of the ‘Pinore’ variety and parental varieties indicated with the OIV descriptors (OIV 2009).

	Ampelographic Characteristics													
Genotype	Young Shoot				Mature Leaf						Bunch		Berry	
(OIV code)	001	004	051	053	067	068	070	076	079	084	204	223	225	231
Pinot Noir’	5	5	1/3	5	3	2	1	2	3/5	3	3	2	6	1
‘Pinore’	5	5	2/3	5	4	3	1	2	5	3	3	2	6	3
‘Regent’	5	5	1/3	5	2	3	2	2	3/5	3	5	1/2	6	1

Young shoot: OIV001—opening of the shoot tip; 5, fully open; OIV004—density of prostrate hairs on the shoot tip; 5, medium; young leaf: OIV051—colour of upper side of blade; 1, green; 2, yellow; 3, bronze; OIV053—density of prostrate hairs between main veins on lower side of blade; 5, medium; mature leaf: OIV067—shape of blade; 2, wedge-shaped; 3, pentagonal; 4, circular; OIV068—number of lobes; 2, three; 3, five; OIV070—area of anthocyanin coloration of main veins on upper side of blade; 1, absent; 2, only the petiolar point; OIV076—shape of teeth; 2, both sides straight; OIV079—degree of opening/overlapping of petiole sinus; 3, open; 5, closed; OIV084—density of prostrate hairs between main veins on lower side of blade; 3, low; bunch: OIV204–—bunch density; 3, loose; 5, medium; berry: OIV223—berry shape; 1, obloid; 2, globose; OIV225—colour of skin; 6, blue black; OIV231—anthocyanin coloration of flesh; 1, none or very weak; 3, weak.

**Table 3:** Mean values ( ± standard deviation) of yield components and grape quality parameters of ‘Pinot Noir’ (reference variety) and new variety ‘Pinore’ grafted on ‘Kober 5BB’ rootstock and own rooted nuclear stock (NS) (2014–2016).

2014–2016	Date of Vintage	Shoots Number	Bunches per Vine	Yield kg/Vine	Bunch (g)	Nr. of Berries per Bunch	Berry (g)	Sugar °Oe	Titr. Acidity (g/L)	pH
Pinore-NS *	22.9 ± 2.65	6.0 ± 0.00 a b	11.7 ± 2.52 b	1.36 ± 0.22 b	117.3 ± 8.79 b	91.7 ± 9.65 a	1.2 ± 0.03 b	89.7 ± 7.57 a	8.73 ± 0.23 a	3.09 ± 0.07 a
‘Pinore’	22.9 ± 2.65	7.0 ± 0.82 a	15.5 ± 3.63 a	2.51 ± 0.18 a	168.7 ± 32.98 a	93.3 ± 11.32 a	1.7 ± 0.04 a	88.7 ± 8.22 a	9.93 ± 0.75 a	3.04 ± 0.02 a
‘Pinot Noir’	22.9 ± 2.65	6.9 ± 0.73 a	14.6 ± 2.76 a	2.38 ± 0.23 a	161.4 ± 26.67 a	95.2 ± 8.95 a	1.6 ± 0.03 a b	90.1 ± 6.27 a	9.80 ± 1.35 a	3.1 ± 0.08 a

* Nuclear stock own rooted; different letters denote significant differences among varieties with a > b (*p* ≤ 0.05).

**Table 4:** Chemical and sensorial characteristics of wine of investigated genotype ‘Pinore’ and the reference cultivar ‘Pinot Noir’ (2014–2016).

Average 2014–2016	Alcohol vol%	Total Extract g/L	Tartaric Acid g/L	Anthocyans mg/L	Color Intensity	Sensory Evaluation
‘Pinore’	12.00 ± 0.76 a	25.3 ± 1.79 a	5.98 ± 1.07 a	1534.4 ± 722.33 a	5.53 ± 0.69 a	17.42 ± 0.183 a
‘Pinot Noir’	12.22 ± 0.59 a	23.6 ± 1.98 b	4.80 ± 0.93 b	70.37 ± 33.21 b	2.45 ± 0.38 b	17.29 ± 0.155 a

Different letters denote significant differences among varieties, with a > b (*p* ≤ 0.05).

## Data Availability

Data are available from the breeder.
